# Simple surgical technique for minimizing the risk of extensor pollicis longus rupture following volar plate fixation of distal radial fracture: A case report

**DOI:** 10.1016/j.ijscr.2022.106869

**Published:** 2022-02-25

**Authors:** Akira Hara, Yasuhiro Yamamoto, Satoshi Ichihara, Masao Suzuki, Yuichiro Maruyama

**Affiliations:** a Hand Surgery Center, Juntendo University Urayasu Hospital, 2-1-1 Tomioka, Urayasu, Chiba 279-0021, Japan; b Department of Orthopedic Surgery, Juntendo University Urayasu Hospital, 2-1-1 Tomioka, Urayasu, Chiba 279-0021, Japan

**Keywords:** Complication, Tendon rupture, Plate fixation, Screw protrusion, Tangential view

## Abstract

**Introduction and importance:**

Extensor pollicis longus (EPL) tendon injury is a major complication in distal radial fracture repair. The risk factors for EPL tendon injury are prominent dorsal screws, direct intraoperative damage through drilling, and/or dorsal roof fragments. Herein, we introduce a simple technique to minimize the risk of EPL tendon rupture after volar plate fixation of distal radial fracture.

**Case presentation:**

The patient was a 67-year-old woman with an intra-articular unstable distal radial fracture treated by volar locking plate fixation. Intraoperatively, we opened the third compartment after screw fixation. Because the screw had penetrated the floor of the third compartment, we moved the EPL tendon out of its groove and closed the third compartment by suturing the retinaculum. We confirmed that the EPL tendon was intact 7 years postoperatively, even though the screw was prominent in the third compartment.

**Clinical discussion:**

After volar plate fixation of the distal radial fracture, we partially open the third compartment through an approximately 2-cm-long incision on the ulnar side of Lister's tubercle. If the screw is prominent in the third compartment, we completely open the third compartment, take the EPL tendon out of its groove, and close the compartment by suturing the retinaculum. Our method was proved useful because the EPL tendon has remained intact for 7 years with the screw protruding into the third compartment.

**Conclusion:**

Our surgical technique is useful to prevent secondary EPL tendon rupture after distal radial plate fixation.

## Highlights

• We describe a simple technique to prevent secondary EPL tendon rupture.
• The EPL tendon is checked by opening the third compartment via a small incision.
• Only an additional 10 minutes are required to check the EPL tendon.
• If there is screw penetration, we remove the EPL tendon and close the compartment.
• If there is no screw penetration, the EPL tendon is left untreated.

## 1 Introduction

Extensor tendon rupture after volar plate fixation remains a major problem in distal radial fracture repair. The most commonly affected tendon is the extensor pollicis longus (EPL) tendon, owing to its confinement within the EPL groove. The reported incidence of EPL tendon rupture following volar plating is 0.29%–5.7% [1], [2], [3], [4]. The risk of delayed EPL tendon rupture is increased in cases with prominent dorsal screws, direct intraoperative damage caused by drilling, and dorsal roof fragments, especially an island-shaped fracture of Lister's tubercle [2], [4], [5]. It is difficult to radiologically assess screw prominence in distal radial fractures because of the complex geometry of the distal radius and the potential for comminuted dorsal fractures. The dorsal tangential view is the only possible in vivo view of the dorsal radial cortex to obtain reliable assessment of the distance between the screw tip and the dorsal cortex [6].

Techniques proposed to avoid extensor tendon injury include the use of unicortical screws and avoidance of dorsal surface penetration [7]; however, these techniques may reduce the mechanical stability of the fracture repair. Therefore, bicortical fixation is sometimes required. The primary goals of fracture fixation and stability must not be compromised in an attempt to minimize the risk of extensor tendon injury.

We introduce a new technique to minimize the risk of EPL tendon rupture after volar plate fixation of distal radial fracture without shortening the screw length or removing the dorsal roof fragment. In brief, this technique comprises opening the third compartment through a small dorsal incision. If the screw has penetrated the dorsal cortex and is prominent in the third compartment, we take the EPL tendon out of its groove and close the compartment by suturing the retinaculum to leave the EPL tendon over the repaired retinaculum. If the screw does not protrude into the third compartment, we leave the EPL tendon in the third compartment. The indications for our technique are patients who present with distal radial fracture treated by volar locking plate with a dorsal roof fragment around Lister's tubercle or with fractures in which the screw might penetrate the dorsal cortex or damage the EPL tendon. In patients with distal radial fracture with a dorsomedial fragment, we drill to penetrate the dorsal cortex and choose a screw that is long enough to fix the unstable dorsomedial fragment.

We report a case in which the EPL tendon was removed from the third compartment because of screw protrusion in the third compartment at the time of plate fixation of a comminuted distal radial fracture with a dorsomedial fragment. We confirmed that the EPL tendon was intact 7 years postoperatively, even though the screw was prominent in the third compartment. The work has been reported in line with the SCARE criteria [8].

## 2 Case report

**Fig. 1:**
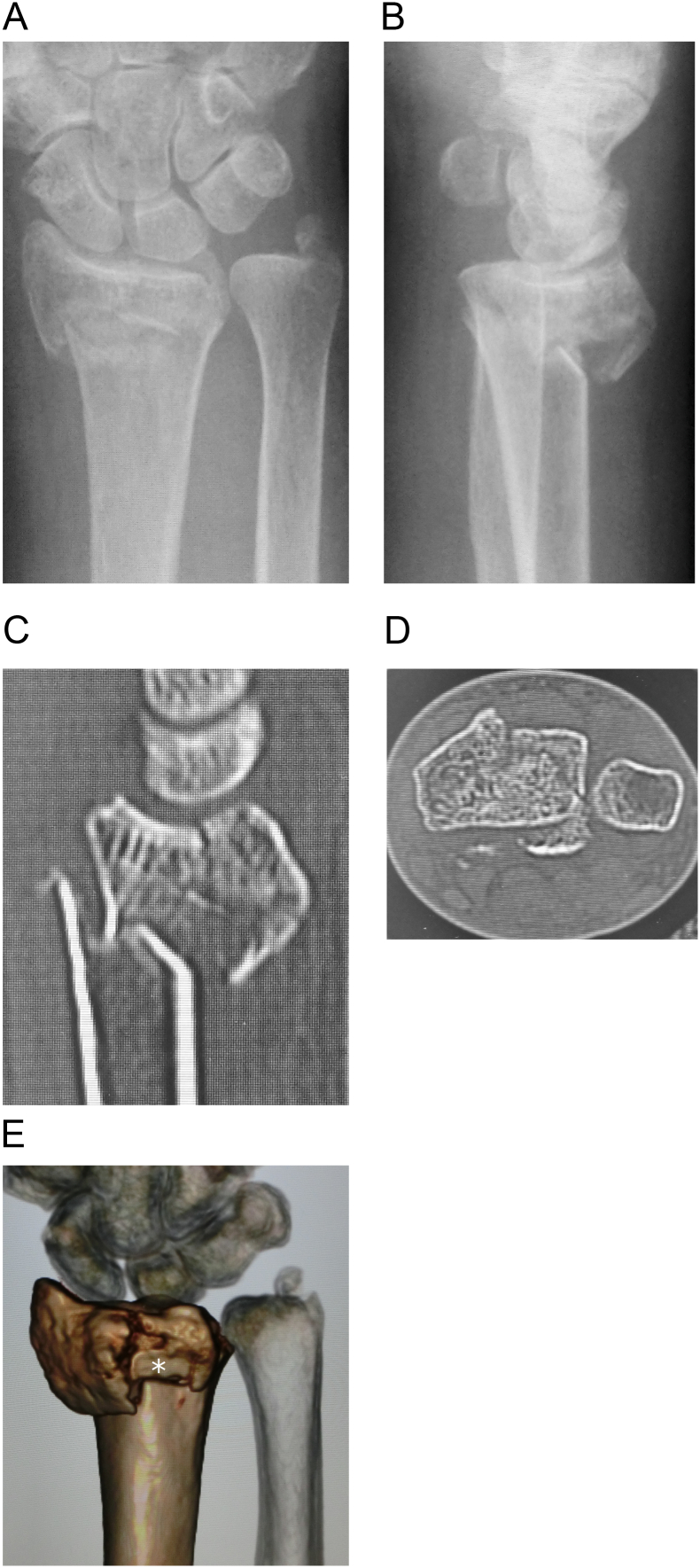
Preoperative image showing the comminuted intra-articular distal radial fracture. A and B: preoperative radiographs, C and D: sagittal and axial views of computed tomography images, E: three-dimensional computed tomography image. The dorsomedial lunate facet fragment and the dorsal roof fragment (white asterisk) are visible.

**Fig. 2:**
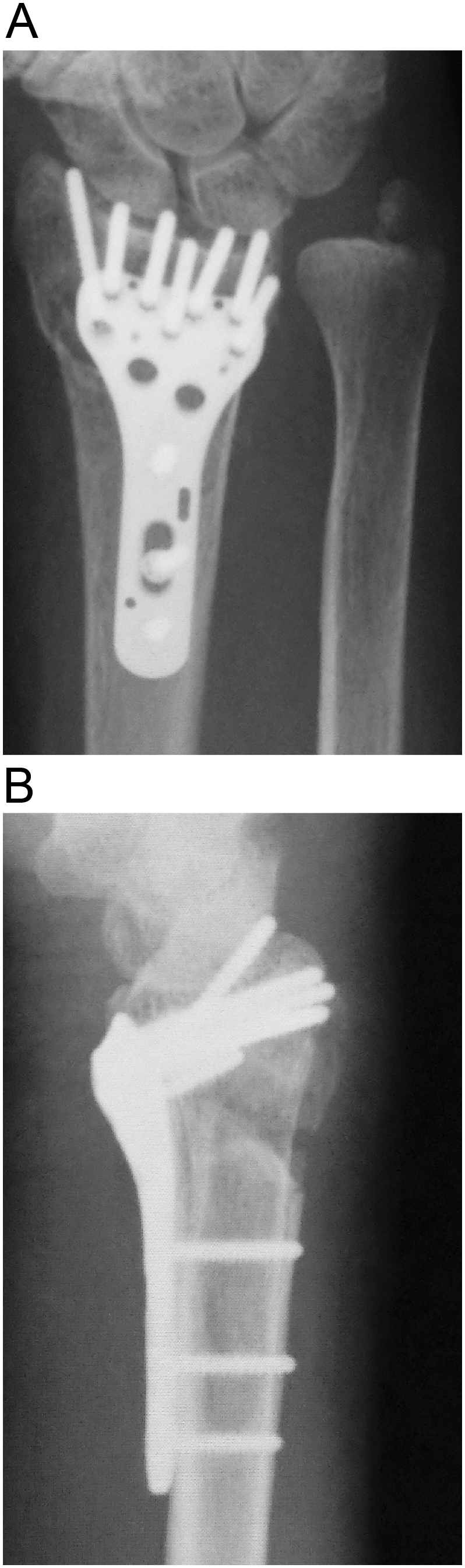
Postoperative radiograph.

**Fig. 3:**
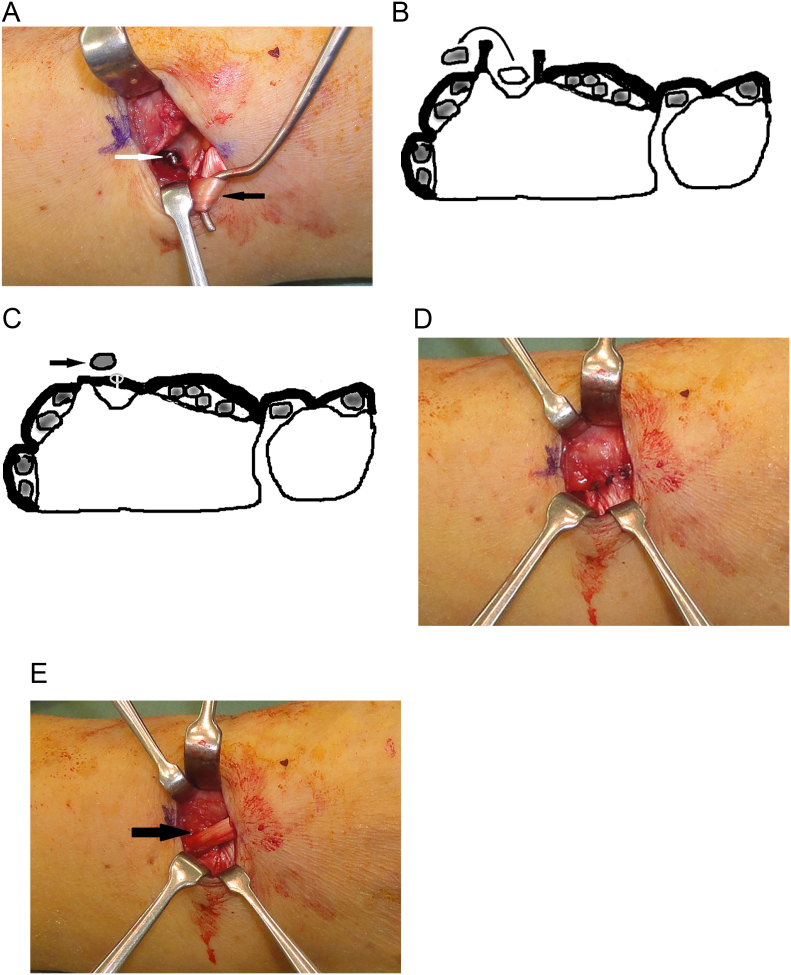
The third compartment is partially opened through a small incision just ulnar to Lister’s tubercle. A: The extensor pollicis longus tendon (black arrow) is retracted out of its groove. The screw (white arrow) is visible penetrating the dorsal cortex into the third compartment. B: The third compartment is completely opened and the extensor pollicis longus tendon is moved out of the compartment. C: The compartment is closed by suturing the retinaculum while the extensor pollicis longus tendon (black arrow) is repositioned over the third compartment. D: The third compartment is closed by suturing the retinaculum. E: The extensor pollicis longus tendon (black arrow) is positioned over the repaired retinaculum.

A 67-year-old woman was referred to our hospital because she sustained an intra-articular unstable distal radial fracture with a dorsomedial fragment on her dominant right side (Fig. 1A-E). She had no history of smoking, diabetes mellitus, or alcohol intake. She could walk without any walking aids. The patient provided informed consent preoperatively for the EPL tendon to be evaluated through a dorsal incision if our intraoperative assessment suggested that the tendon was likely to be injured. We treated this fracture using a volar locking plate system (Stellar; HOYA Technosurgical, Inc., Tokyo, Japan). Intraoperatively, we drilled to penetrate the dorsal cortex and chose a screw that was long enough to catch the dorsal cortex, as the dorsomedial fragment was unstable (Fig. 2). The operation was performed in our institution. The procedure was started by the one of the authors (M.S., a junior trainee with 5 years of surgical specialty training). The operator was then changed to the first author (A.H., a highly experienced specialist surgeon), who opened the third compartment after screw fixation. As the screw had penetrated the third compartment and was prominent (Fig. 3A), we completely opened the third compartment and moved the EPL tendon out of its groove (Fig. 3B). We then closed the third compartment by suturing the retinaculum (Fig. 3C, D) and left the EPL tendon positioned over the repaired retinaculum (Fig. 3E). After the operation, the patient attended our outpatient clinic until bone union was achieved. The patient did not want the hardware removed.

**Fig. 4:**
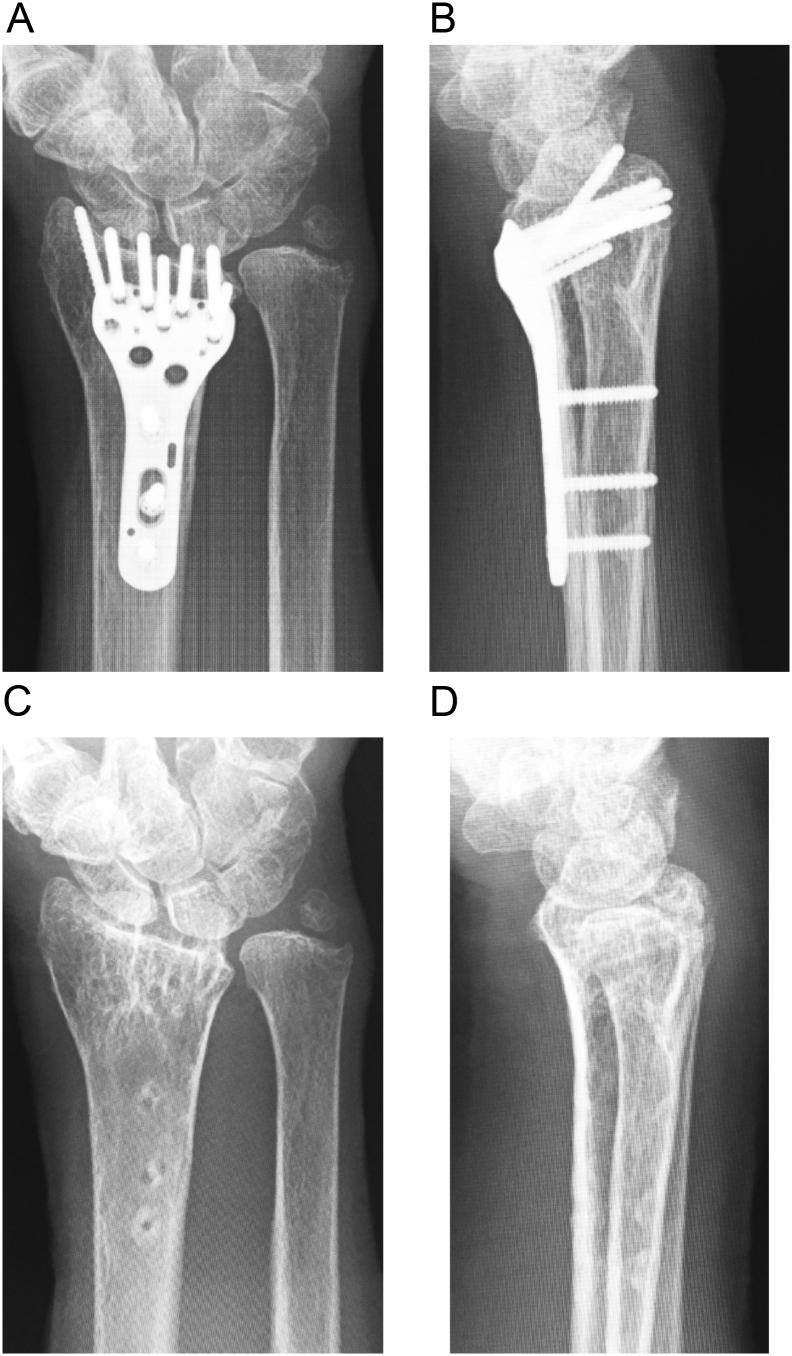
Radiographs of the distal radius at 7 years after fracture repair. A and B: preoperative images, C and D: after hardware removal.

**Fig. 5:**
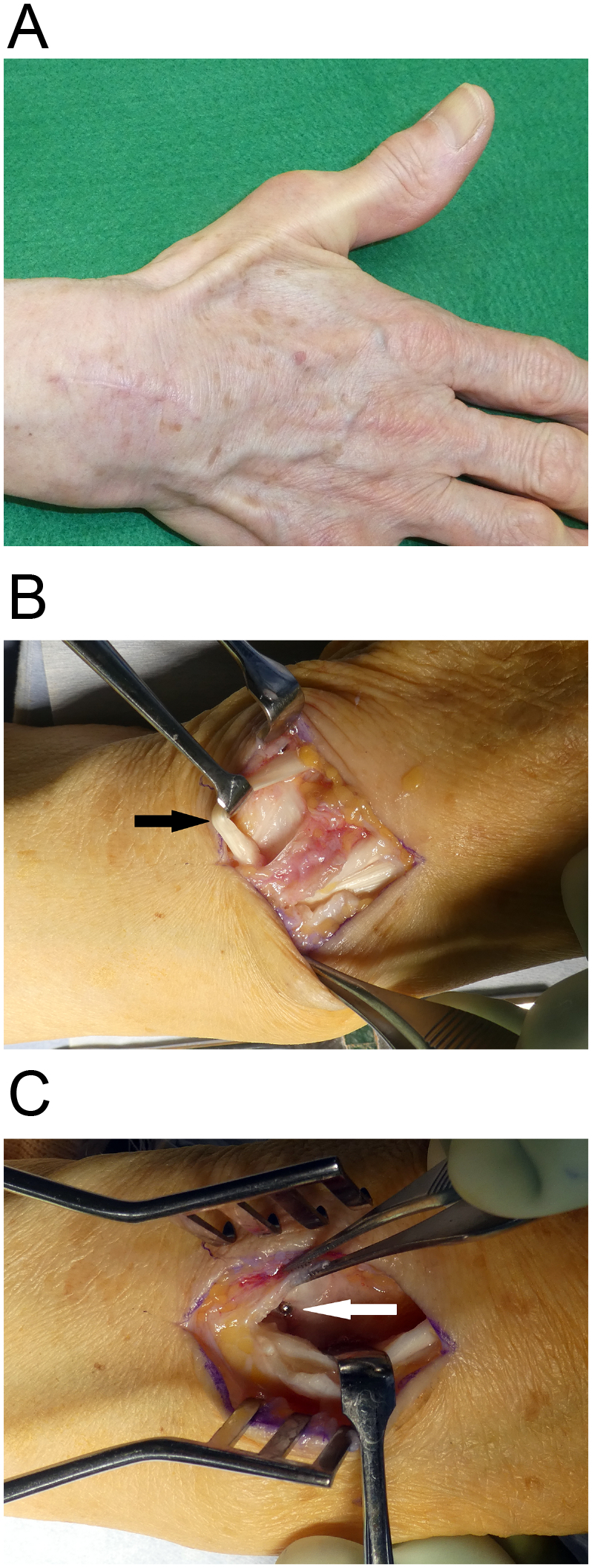
Images obtained 7 years after fracture repair. A The patient is able to extend her thumb without bowstringing. B Intraoperatively, we made a dorsal incision to confirm that the extensor pollicis longus tendon (black arrow) is located outside the third compartment and is intact. C Screw penetration (white arrow) is visible at the base of the third compartment when the common digital extensor tendon is retracted. The plate and screws are removed.

Seven years postoperatively, the patient returned to our hospital because of osteoporosis. She used her right hand as usual without disability. X-ray images showed a healed fracture with dorsal protrusion of the distal locking screws (Fig. 4A, B). The patient could fully extend her thumb, and there was no obvious bowstringing of the EPL tendon (Fig. 5A). In accordance with our recommendation, the patient consented to have the hardware removed and the extensor tendons examined. The operation was performed in our institution by the first author (A.H.). Intraoperatively, we checked the EPL tendon through a dorsal incision and partially opened the third and fourth compartments. The EPL tendon was located outside the third compartment and positioned as it had been in the previous operation, with no irritation of the tendon (Fig. 5B). We confirmed that the screw penetrated into the third compartment when the common digital extensor tendon was retracted (Fig. 5C). Finally, we repaired the retinaculum and removed the hardware (Fig. 4C, D). At the final assessment performed 2 months after the removal of the hardware, the patient had no pain and could fully extend the thumb. The grip strength was 100% compared with the contralateral side. The Mayo wrist score was 100 points, and the Quick Disabilities of the Arm, Shoulder and Hand score was 2.27.

## 3 Discussion

In patients with dorsal bone comminution or gaps after reduction and volar plate application for distal radial fracture, Benson et al. recommend open assessment of the third extensor compartment via a small dorsal incision to check for possible sources of injury to the EPL tendon [5]. Furthermore, Cha et al. reported that an island-shaped Lister's tubercle fracture is associated with a high rupture risk, as callus formation narrows the EPL groove [2]. They recommend the removal of such a high-risk fragment by combining volar plating with a dorsal approach, so that the EPL is seated on the repaired periosteum after the removal of the free fragment [2].

In our method, after volar plate fixation of the distal radial fracture, we partially open the third compartment through an approximately 2-cm-long incision on the ulnar side of Lister's tubercle. We directly identify the EPL tendon and the floor of the third extensor compartment by gently retracting the EPL tendon. If the screw penetrates the dorsal cortex into the third compartment or there is a dorsal roof fragment, we do not perform an intraoperative screw change or fragment resection, but instead completely open the third compartment and take the EPL tendon out of its groove (Fig. 3B). We then close the compartment by suturing the retinaculum while the EPL tendon is repositioned over the third compartment (Fig. 3C). If the screw does not protrude into the third compartment, we leave the EPL tendon in the partially opened third compartment.

Our surgical technique makes it easy to check for EPL tendon injury under direct visualization, which only takes an additional 10 min. If the EPL tendon is injured, it can be repaired directly. This procedure is useful to prevent secondary EPL tendon rupture after distal radial plate fixation. EPL tendon bowstringing may occur, but did not occur in our case.

## 4 Conclusions

We experienced a case in which the EPL tendon was intact 7 years postoperatively, even though the screw used to fix the volar plate was prominent in the third compartment. Our method minimizes the risk of EPL tendon rupture following volar plate fixation of a distal radial fracture.

## Sources of funding

None.

## Ethical approval

None.

## Consent

Written informed consent was obtained from the patient for publication of this case report and accompanying images. A copy of the written consent is available for review by the Editor-in-Chief of this journal on request.

## Availability of data and material

Not applicable.

## Research registration

None.

## Author contributions

Akira Hara and Yasuhiro Yamamoto conducted a literature search and drafted the manuscript.

Akira Hara and Masao Suzuki performed the operation.

Akira Hara and Satoshi Ichihara contributed during the patient management and participated in the design of the case report and coordination and helped draft the manuscript.

Akira Hara wrote up.

Yuichiro Maruyama was consultant involved in management of patient, main guidance for write up.

## Registration of research studies

None.

## Guarantor

Akira Hara.

## Provenance and peer review

Not commissioned, externally peer-reviewed.

## Declaration of competing interest

None.
